# A Prospective Evaluation of Percutaneous Vertebroplasty in Osteoporotic Vertebral Compression Fracture Patients

**DOI:** 10.7759/cureus.40255

**Published:** 2023-06-11

**Authors:** Yasum Litin, Shivani Rastogi, Anurag Agarwal, Shilpi Misra, Pankaj Agrawal, Deepak Malviya

**Affiliations:** 1 Department of Anaesthesiology, Dr Ram Manohar Lohia Institute of Medical Sciences, Lucknow, IND; 2 Department of Orthopaedics, Dr Ram Manohar Lohia Institute of Medical Sciences, Lucknow, IND

**Keywords:** osteoporosis, chronic low back pain (clbp), osteoporotic vertebral compression fracture (ovcf), quality of life (qol), percutaneous vertebroplasty

## Abstract

Background

Osteoporotic vertebral compression fracture (OVCF) is a common problem in old age, which causes incapacitating pain and leads to significant disability, morbidity, and mortality. Percutaneous vertebroplasty (PVP), a minimally invasive procedure, results in immediate pain relief with decreased morbidity. The primary aim of this study was to evaluate the quality of life (QOL), as denoted by the Roland-Morris Disability Questionnaire (RMDQ) score. In contrast, the secondary aims were determining pain relief using the 11-point Numeric Pain Rating Scale (NPRS) and vertebral height restoration and wedge angle measurements after PVP.

Methodology

This prospective, longitudinal, interventional study on the efficacy of PVP was conducted among patients with low back pain due to osteoporotic vertebral collapse in a tertiary care institute. Patients with OVCF were managed by PVP and followed at one week, one month, three months, and six months for improvement in QOL by the RMDQ score and pain relief using the NPRS. The pre and post-vertebroplasty wedge angle and vertebral height (anterior, middle, and posterior columns) at one week and six months were also compared by pre and post-vertebroplasty lateral view skiagrams.

Results

A total of 24 patients were included in this study based on the inclusion and exclusion criteria. The demographic data were comparable. The RMDQ score showed a statistically significant difference in post-PVP at one week (p = 0.044), one month (p = 0.031), three months (p = 0.022), and six months (p = 0.018). There was a statistically significant difference in the NPRS at six months compared to the pre-PVP status, showing drastic pain relief in patients after PVP. The mean wedge angle (20.5 ± 2.07) measurement was reduced with a statistically significant increase in anterior body height restoration from pre-PVP to six months. There was no significant change in height at the middle and posterior columns compared to the pre-PVP height.

Conclusions

PVP is an effective, safe, minimally invasive pain and spine intervention for OVCFs with improved QOL and restoration of vertebral height.

## Introduction

Osteoporosis is a systemic skeletal disorder characterized by low bone mass and micro-architectural deterioration, resulting in bone fragility and increased fracture risk. The World Health Organization (WHO) defines osteoporosis as having a bone mineral density (BMD) greater than 2.5 standard deviations below the young normal adult reference population at the hip or lumbar spine. Osteoporotic vertebral fracture is a common problem in old age, which causes incapacitating pain and leads to significant morbidity, disability, and mortality. It is estimated that over 200 million people have osteoporosis worldwide [1]. Of the 230 million population of India, around 50 million people have osteoporosis [2]. According to the European Vertebral Osteoporosis study, women are more prone to develop vertebral deformity than men, increasing exponentially with age, i.e., from 5% to 25% in women and from 10% to 18% in men aged 50 years and 75 years, respectively [3]. The traditional conservative treatment of compression fractures includes bed rest, bracing, lifestyle modification, non-steroidal anti-inflammatory agents, and anti-osteoporotic treatment. This regimen is successful in about two-thirds of patients but can be improved.

Bed rest and pain may lead to reduced activity and immobility, which may cause further bone loss and other problems such as atelectasis and deep vein thrombosis, thus decreasing quality of life (QOL). According to the previous literature, after about 12 weeks of conservative therapy, 85% of symptomatic patients get relief from pain and discomfort produced by these acute osteoporotic fractures. The remaining 15% of patients with chronic osteoporotic compression fractures who do not respond to conservative therapy may require open surgery or percutaneous vertebroplasty (PVP) [4,5]. PVP stabilizes osteoporotic vertebral compression fractures (OVCFs) by injecting bone cement polymethylmethacrylate (PMMA) into the spine. The cement solidifies, preventing further vertebral body collapse and supporting the trabeculae’s microfractures. Restoring the vertebral height, alignment, and wedge angle relieves pain [6]. Alignment and height restoration improve deformities, spinal dynamics, functional outcomes, post-procedure fractures [7], and QOL and pain [8].

PVP has a rare complication rate of about 1.6% to 2.8%, which includes complications associated with needle placement, infection, cement extravasation, hemorrhage, cement penetration of the nerve root foramen or spinal canal resulting in radiculopathy or spinal cord compression, and embolic events due to marrow fat or cement entering the circulation. Only a few studies have correlated the restoration of the vertebra’s height and functional outcomes after PVP. Thus, this study evaluated the QOL and functional outcomes after PVP and compared the pre- and post-vertebroplasty wedge angles and vertebral heights (anterior, middle, and posterior).

## Materials and methods

**Figure 1:**
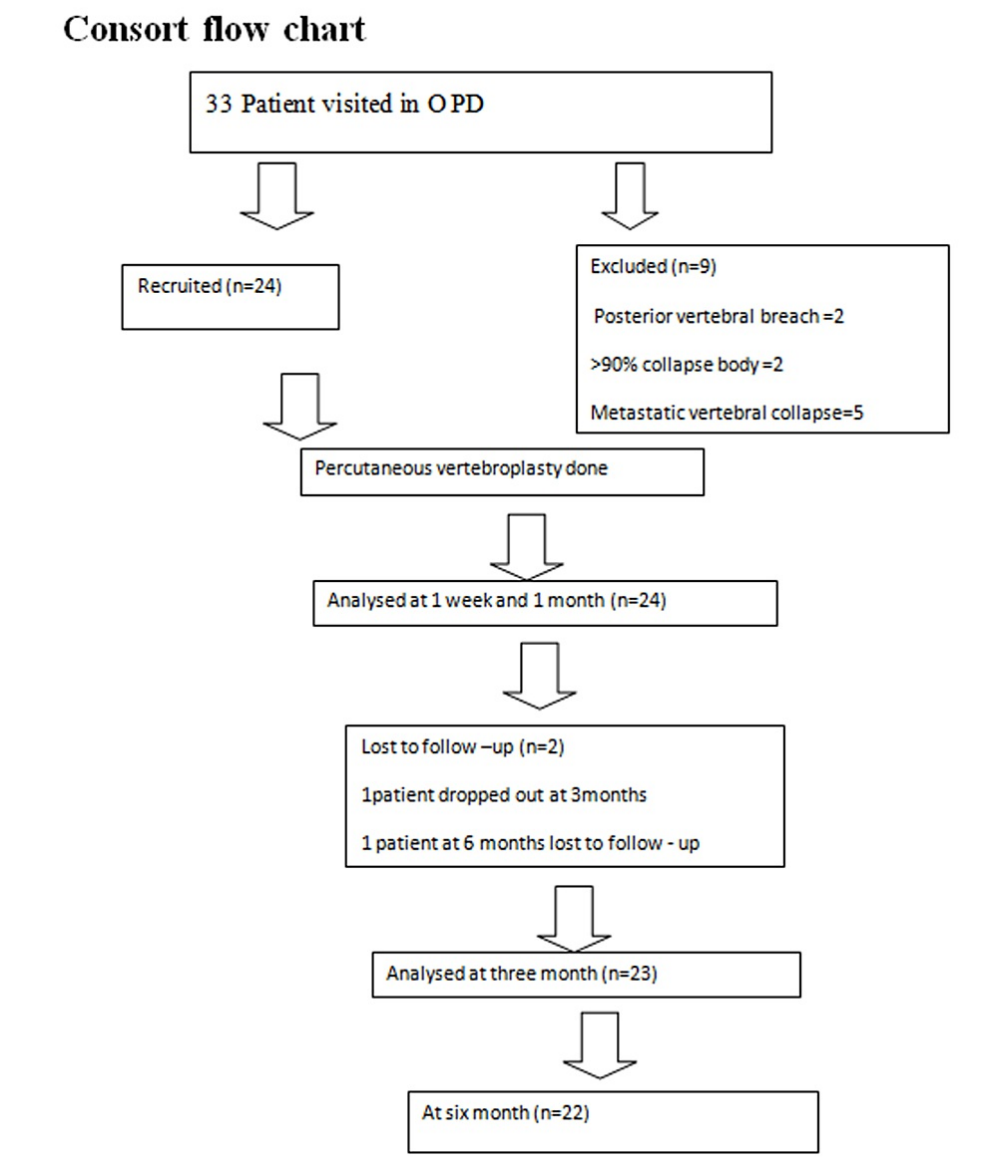
CONSORT flow chart.

This is a prospective, longitudinal, interventional study. After approval from the Institutional Ethical Committee (IEC NO. 70/19) and Clinical Trials Registry in India (CTRI/2020/08/027343), 24 OVCF patients with failed conservative care and chronic back pain (>12 weeks) who were treated with PVP from September 2019 and March 2022 were included in this study. Inclusion criteria included age between 18 and 80 years and a normal coagulation profile. Patients with a refusal of consent; those with pedicle fracture, cord compression, local (osteomyelitis, discitis) or systemic infection, concomitant hip fractures, osteoporotic vertebral collapse >90%, previous vertebroplasty, posterior vertebral body breach, and any significant compromise of the spinal canal by retropulsion bone fragment or tumor; pregnant patients; vertebral compression fracture patients with multiple myeloma and metastatic tumor; or patients with known coagulopathy were excluded (Figure 1).

A previous study has shown that the Roland-Morris Disability Questionnaire (RMDQ) scores were generally distributed in OVCF patients treated with vertebroplasty, with a mean of 19 and a standard deviation (SD) of 5. We intended to measure a minimum reduction in the RMDQ score of 4 points that would be clinically significant. To attain these objectives, we needed to enroll a minimum of 15 patients to be able to reject the null hypothesis that the mean reduction in the RMDQ score is zero with a type I error of 0.05 and a power of 0.8.

n = (Z1 − 𝖺 + Z1 − 𝛽)2 𝜎2

µ1 − µ2

at 95% confidence interval (CI) = 1.96

Z1 − 𝛽 at 80% power = 0.84

𝜎 = SD

µ1, µ2 = means

µ1 − µ2 = assumed difference in mean

= 15 (calculated sample size)

A total of 24 patients were included in this study.

After written informed consent and preoperative evaluation, all patients underwent PVP in a dedicated pain medicine operation theater. After securing intravenous access and institution of essential monitoring (pulse and non-invasive blood pressure monitoring, electrocardiography monitoring, and oxygen saturation monitoring), all patients were placed in a prone position with pillows under the chest and pelvis to increase the lumbar lordosis. PVP was conducted under monitored anesthesia care with sedation and local anesthesia.

Under fluoroscopic guidance, the target vertebra was identified in the anteroposterior (AP) view, and the entry point was marked at the superior lateral quadrant of the target pedicle. An 11-gauge bone biopsy needle (Jamshidi needle) was inserted transpedicular and advanced under AP fluoroscopic control in an antero-medio-caudal direction with rotatory hand pressure or a mallet. The Jamshidi needle path, which was parallel to the superior and inferior edges of the pedicle, was ensured by intermittent lateral fluoroscopy and advanced into the anterior third portion of the vertebra as this area is devoid of venous plexuses. In a bilateral technique, both Jamshidi needles were inserted before the cement was injected from both sides simultaneously. After satisfactory placement of Jamshidi needles, the vertebroplasty-specific cement consisting of PMMA polymer was mixed with PMMA monomer to obtain the toothpaste consistency of bone cement. Subsequently, under lateral real-time fluoroscopic control, PMMA cement was injected to ensure early detection of cement leaking into the epidural, vena cava, or disc space until it reached the posterior vertebral wall. After the cement had been injected, the Jamshidi needles were removed after confirmation in lateral view. Following needle removal, patients were advised to rest for two hours and were mobilized.

Observational indexes and evaluation criteria

**Figure 2:**
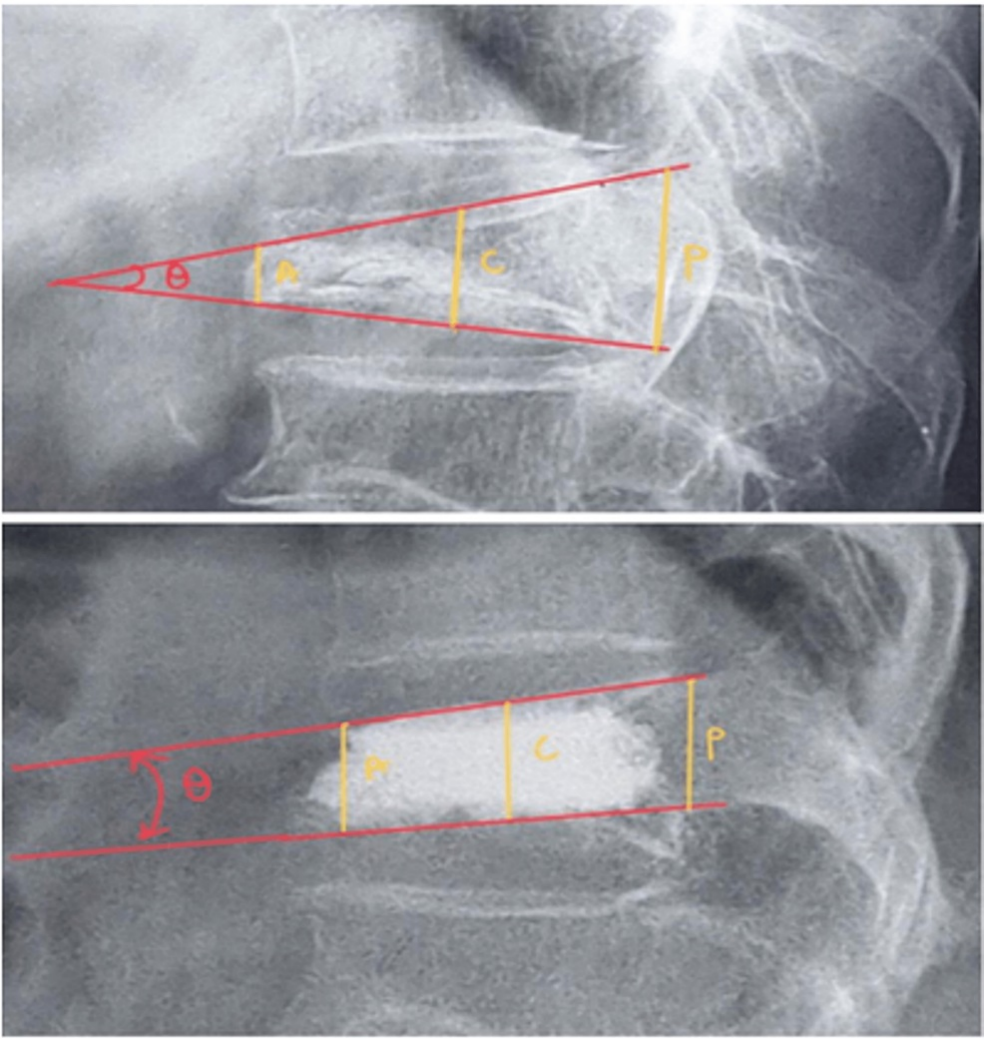
Measurement of the collapsed vertebral body. Vertebral body height anterior border (A), center (C), posterior border (P), and wedge angle (Ꝋ) were measured.

After PVP, patients were observed for two hours in the postoperative room and then made ambulatory and were discharged in the evening with instructions for the follow-up visit at one week, one month, three months, and six months. At every follow-up visit, patients were evaluated for QOL by the RMDQ (on a scale of 0 to 23, with higher scores indicating a more significant disability) and for pain relief by the 11-point Numeric Pain Rating Scale (NPRS). Lateral static X-rays were used to measure wedge angle (the angle between the superior endplate line and the inferior endplate line of the fractured vertebral body) and restoration of vertebral height (measured by comparing pre and postoperative lateral view X-rays) (Figure 2). Any complications during or after the PVP were recorded.

The collected data were compiled using a Microsoft Excel spreadsheet. Statistical analysis was done using SPSS version 21.0 (IBM Corp., Armonk, NY, USA). Demographic data were analyzed with the difference in mean values, and the chi-square test was used for categorical variables. The demographic data were presented as mean and SD, and non-parametric data were analyzed by the chi-square test. The numerical data were analyzed by parametric and non-parametric tests based on whether they were distributed normally or non-normally. Independent Student’s t-test was used for testing RMDQ, NPRS, vertebral height (anterior, middle, and posterior border), and wedge angle. P-values <0.05 were considered significant with a 95% CI in the study.

## Results

A total of 24 patients were included in the study. As two patients were unable to attend the outpatient department after three months, their follow-up was done telephonically.

The demographic data regarding age, sex, and duration of pain were homogenous in the study group. Of the 24 patients, 19 (79.16%) were females, and five (21.7%) were males. The mean age ± SD was 62.45 ± 9.5 years, with an age range of 50-79 years. A total of 37 collapsed vertebrae in 24 patients were treated by PVP. Out of the 37 treated vertebrae, 24 were treated by a unipedicular approach, while 13 were treated by a bi-pedicular approach (Table 1). The cement volume used was about 4.42 ± 1.0036. There was minor cement leakage in 11 vertebrae (29.7%), and six (25%) patients complained of mild pain over the local (needle insertion) site, which was relieved by analgesics. There were no new adjacent vertebral fractures in the six-month follow-up.

**Table 1:** Details of vertebrae treated and complications.

Demographic details	Mean ± SD
Cement volume (mL)	4.42 ± 1.0036
Approach (vertebra)	
Uni-pedicular	24 (64.8%)
Bi-pedicular	13 (35.1%)
Cement leakage	11 (29.7%)
Back pain	6 (25%)

The QOL RMDQ score showed consistent and statistically significant improvement after PVP at every follow-up (mean ± SD) from 20.26 ± 2.91 pre-PVP which reduced to 16.39 ± 3.17 at one week (p = 0.044), 11.39 ± 2.4 at one month (p = 0.031), 8.86 ± 2.98 at three months (p = 0.022), and 8.43 ± 3.6 at six months (p = 0.018) (Table 2).

**Table 2:** Quality of life in terms of RMDQ score (pre-PVP and post-PVP). PVP = percutaneous vertebroplasty; SEM = standard error of the mean; df = degree of freedom; NS = non-significant; S = significant; RMDQ = Roland-Morris Disability Questionnaire

Period	Mean ± SD	SEM	t	df	P-value
Pre-PVP	20.26 ± 2.91	0.607	33.37	22	0.061 (NS)
One week	16.39 ± 3.17	0.661	24.77		0.044 (S)
One month	11.39 ± 2.4	0.024	18.23		0.031 (S)
Three months	8.86 ± 2.98	0.623	14.22		0.022 (S)
Six months	8.43 ± 3.6	0.0.761	11.07		0.018 (S)

The mean ± SD NPRS at pre-procedural was 9.17 ± 1.30 (p = 0.134), which reduced to 5.34 ± 1.61 (p = 0.033), 2.91 ± 1.23 (p = 0.021), 1.73 ± 1.25 (p = 0.014), 1.65 ± 1.30 (p = 0.001) at one week, one month, three months, and at six months, respectively. There was a highly statistically significant difference in the NPRS at six months, showing drastic pain relief in the patients after PVP (Table 3).

**Table 3:** Pain relief in terms of NPRS in the study population (pre-PVP and post-PVP). PVP = percutaneous vertebroplasty; SEM = standard error of the mean; df = degree of freedom; NS = non-significant; S = significant; NPRS = Numeric Pain Rating Scale

Period	Mean ± SD	SEM	t	df	P-value
Pre-PVP	9.17 ± 1.30	0.271	33.71	22	0.134 (NS)
One week	5.34 ± 1.61	0.336	15.90		0.033 (S)
One month	2.91 ± 1.23	0.258	11.26		0.021 (S)
Three months	1.73 ± 1.25	0.260	6.67		0.014 (S)
Six months	1.65 ± 1.30	0.271	6.09		0.001 (S)

**Figure 3:**
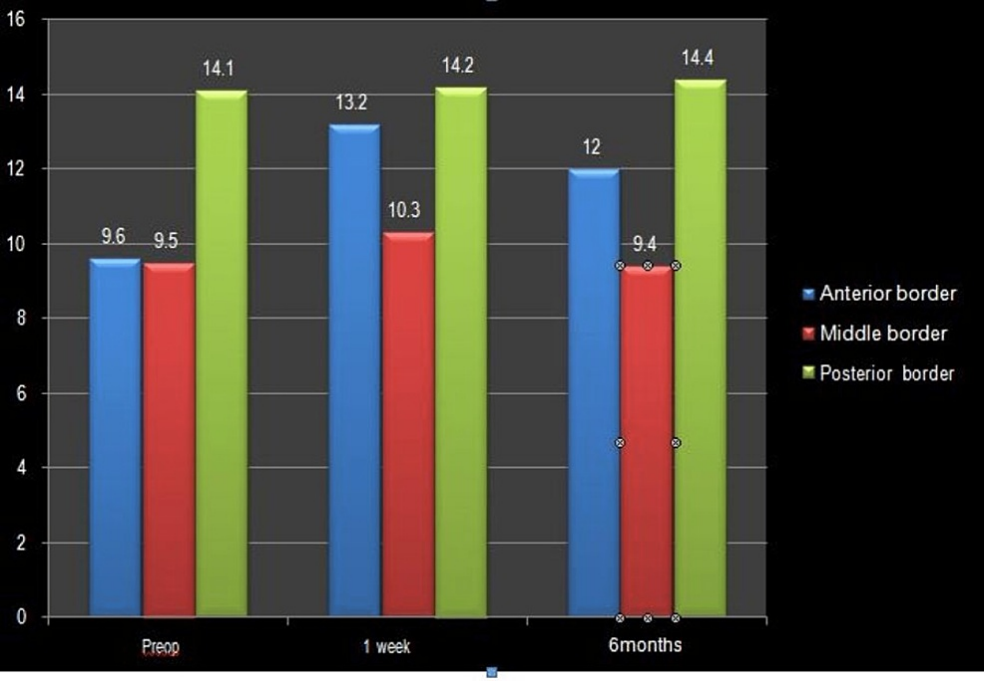
Representation of vertebral height (anterior, middle, and posterior border).

The pre-procedural mean ± SD was 9.6 ± 0.238 (p = 0.057), followed by 13.2 ± 0.29 (p = 0.033) at one week, which decreased to 12 ± 0.47 (p = 0.024) at six months. An increase in the anterior border measurement suggested a statistically significant increase in anterior vertebral height after PVP (Table 4, Figure 3).

**Table 4:** Vertebral height (anterior border pre-PVP and post-PVP in mm). PVP = percutaneous vertebroplasty; SEM = standard error of the mean; df = degree of freedom; NS = non-significant; S = significant

Period	Mean ± SD	SEM	t	df	P-value
Pre-PVP	9.6 ± 02.38	0.049	19.51	22	0.057 (NS)
One week	13.2 ± 02.9	0.061	21.5		0.033 (S)
Six months	12 ± 04.7	0.098	12.14		0.024 (S)

The mean wedge angle (20.5 ± 2.07) measurement reduced from pre-PVP to one week (12.31 ± 1.70) and six months (11.9 ± 3.10). A statistically significant difference between one week and six months (p ≤ 0.05) showed a reduction in pain intensity with a reduction in wedge angle after PVP (Table 5).

**Table 5:** Wedge angle (Pre-PVP and post-PVP). PVP = percutaneous vertebroplasty; SEM = standard error of the mean; df = degree of freedom; NS = non-significant; S = significant

Period	Mean ± SD	SEM	t	df	P-value
Pre-PVP	20.5 ± 2.07	0.43	47.65	21	0.18 (NS)
One week	12.31 ± 1.70	0.36	33.96		0.015 (S)
Six months	11.9 ± 3.10	0.66	18.01		0.011 (S)

## Discussion

An OVCF is a common cause of debilitating back pain in elderly patients. PVP is the most widely used and standard minimally invasive method for treating OVCF owing to its simple technique, minimal morbidity, better therapeutic effect, and speedy recovery.

Of 24 patients in our study, 19 (79.16%) were female. Previous studies also showed that women accounted for 72% of patients with OVCF [9]. Age significantly impacts the severity of osteoporosis, as bone mass loss increases with age [10]. The mean age in our study was 62.45 ± 9.5 years.

PVP can be done via a bi-pedicular or unipedicular approach. The unipedicular approach has several advantages, such as being less time-consuming and having fewer complications. Of the 37 treated vertebrae, 24 (64.8%) were treated using a unipedicular approach as the PMMA flowed across the midline and adequately filled both vertebral hemispheres. The remaining 13 (35.1%) vertebral bodies were treated via a bipedicular approach (Table 1).

According to earlier research, the thoracic and thoracolumbar levels required 4 mL of PMMA to restore stiffness, while the lumbar region required 6 mL. We used an average of 4.42 ± 1.0036 mL cement volume, 3-4.5 mL in thoracic vertebrae, and 4.5- 6.5 mL in lumbar vertebrae (Table 1). The volume requirement was higher in the lumbar vertebra and the bi-pedicular approach. Leakage was higher when the volume of cement used was greater than 6 mL. Sun et al. [11] found that 4-6 mL of PMMA reduced pain immediately, and increased cement volume would also increase the likelihood of cement leakage.

Our study population found 46 OVCFs, of which PVP treated 37 vertebrae. The most commonly involved and treated levels were the thoracolumbar junction (D12-L2). Takegami et al. [12] identified vertebral fractures in 119 vertebrae (20.4%) of the 584 vertebral bodies and reported that the L1 vertebral body had the most vertebral fractures, while the L3 vertebral body had the least.

The RMDQ (Table 2), which consisted of 24 questions about dysfunctions in everyday activities experienced by patients with back pain, was used as the primary outcome measure. The mean RMDQ score value was 20.26 ± 2.91 at pre-PVP, showing significant functional compromise, which significantly reduced post-procedure at one week to 16.39 ± 3.17 (p = 0.044), 11.39 ± 2.4 (p = 0.031) at one month, 8.86 ± 2.98 (p = 0.022) at three months, and 8.43 ± 3.6 (p = 0.018) at six months after PVP. The improvement from baseline was statistically significant (p < 0.018) at all times. Our results regarding RMDQ scores echo the famous Vertos II by Klazen et al. [13] study, which found a mean reduction in RMDQ scores after PVP. Many other studies also found statistically significant improvement in mean RMDQ score after PVP in favor of the PVP group against conservative treatment six weeks showed 87.1% improvement compared to the pre-procedural RMDQ score and the difference was significant (p = 0.0001) [14,15].

The secondary outcome measure was pain relief using the 11-point NPRS. According to numerous case series and research, pain alleviation is expected in 24 hours following PVP. Our patients also experienced statistically significant pain relief following PVP (Table 3). Clark et al. and Cyteval et al. [16,17] also found immediate and profound pain relief after PVP in more than 70% of patients.

Spinal radiographs (anteroposterior and lateral views) were obtained at preoperative, one week, and six months to assess vertebral body height (anterior, middle, posterior) and wedge angle. The fractured vertebral body’s anterior, middle, and posterior vertebral height was measured in mm. In our study, the pre-PVP mean ± SD of anterior height was 09.6 ± 02.38 which increased to 13.2 ± 02.9 at one week but settled at 12 ± 04.7 at six months. The slight decrease in height from one week to six months may be due to cement dispersion with time and load on the vertebra (Figure 3, Table 4).

The non-significant change in middle and posterior vertebral height signifies that most OVCFs are wedge compression fractures which mainly affect the anterior vertebral column. During PVP, cement is deposited in the anterior third of collapsed vertebra. In 72 of 85 treated vertebral bodies, Hiwastashi et al. [18] found that the height of the vertebral bodies had increased. Thirty-three vertebrae (25 patients) increased height by less than 3 mm, while 39 vertebrae (24 patients) increased by more than 3 mm.

The mean wedge angle (20.5 ± 2.07, pre-PVP) was reduced from preoperative to one week (12.31 ± 1.70; p = 0.015) and six months (11.9 ± 3.10; p = 0.011), showing a reduction in the intensity of pain with a reduction in wedge angle after PVP. Wedge angles can predict the outcome of PVP and are desirable to restore maximal alignment. According to some previous studies, improvement in wedge angles after PVP has been reported to be 6° and 3.5° from the pre-procedural, similar to our study [19]. Khurjekar et al. [20] reported that the mean wedge angle improved by 13% from 6° to 5° in 24 and 10 patients, and the preoperative wedge angle was <7° and ≥7°, respectively (Table 5).

There was a small cement leakage in the disc in 11 (29.7%) and no cement leakage in the remaining 26 (70.3%) treated vertebral bodies (Table 1). There was no spinal canal cement leakage in any of our patients. Using toothpaste consistency cement slowly under live lateral fluoroscopic inspection can lower the risk of cement leakage. No adjacent fracture was seen in our study during six months of follow-up. Komemushi et al. [21] reported that the risk of new VCFs was 4.6 times higher in patients with intradiscal cement leakage than in individuals who did not have intradiscal cement leakage. According to Sun et al. [22], the leakage of bone cement from the fractured vertebra’s upper and lower endplates into the intervertebral disc produces more significant stress on the adjacent vertebral body, eventually leading to the adjacent vertebral body fracture. Though there was an intra-discal leakage in 11 (29.7%) of our patients, we still did not encounter new adjacent vertebral fractures during six months of follow-up, which may be due to small leakage, conservative amounts of cement used for vertebroplasty, and active treatment of osteoporosis after PVP.

## Conclusions

PVP is an effective minimally invasive intervention to improve the QOL and immediate pain relief in OVCF patients with a low complication rate. The improvement in the wedge angle and restoration of vertebral height signifies successful outcomes. A slight cement leak does not increase the chances of adjacent vertebral fracture for up to six months at least.
